# Early antimicrobial stewardship team intervention on appropriateness of antimicrobial therapy in suspected sepsis: a randomized controlled trial

**DOI:** 10.1093/jacamr/dlab097

**Published:** 2021-08-27

**Authors:** Zohal Rashidzada, Kelly A Cairns, Trisha N Peel, Adam W Jenney, Joseph S Doyle, Michael J Dooley, Allen C Cheng

**Affiliations:** 1 Pharmacy Department, Alfred Health, Melbourne, Australia; 2 Faculty of Pharmacy and Pharmaceutical Sciences, Monash University, Melbourne, Australia; 3 Department of Infectious Diseases, Alfred Health, Melbourne, Australia; 4 Department of Infectious Diseases, Monash University, Melbourne, Australia; 5 Burnet Institute, Melbourne, Australia; 6 Department of Public Health and Preventative Medicine, Monash University, Melbourne, Australia

## Abstract

**Objectives:**

There has been concern that the imperative to administer rapid antimicrobials in septic patients may result in inappropriate antimicrobial use. We aimed to determine the impact of early antimicrobial stewardship (AMS) team intervention in patients with Medical Emergency Team (MET) calls for suspected sepsis.

**Methods:**

We performed a randomized controlled trial of non-ICU inpatients who had a MET call for suspected sepsis. Patients were randomized to standard care (management of antimicrobial therapy by the treating team) or early targeted intervention (AMS review 48 h post-MET call). The primary outcome was appropriateness of antimicrobial therapy 72 h post-MET call, as determined by a panel of blinded infectious diseases physicians.

**Results:**

In total, 90 patients were enrolled; 45 were randomly allocated to the intervention group, and 45 to the control group. More patients in the AMS intervention group were receiving appropriate antimicrobials 72 h following the MET call (67% versus 44%, *P* = 0.03). In the intervention group, 27 recommendations were made by the AMS team; 74% of recommendations were accepted, including 30% of cases where antimicrobials were discontinued or de-escalated. There were non-significant differences in total duration of antimicrobial therapy (8.7 versus 10.7 days, *P* = 0.39), sepsis-related ICU-admission rates (13% versus 18%, *P* = 0.56) and sepsis-related in-hospital mortality (7% versus 9%, *P* = 0.71) between intervention and control groups, respectively.

**Conclusions:**

AMS team intervention resulted in significant improvement in appropriateness of antimicrobial therapy following MET calls due to suspected sepsis. Targeted AMS review should be implemented to support early antimicrobial de-escalation and optimization in patients with suspected sepsis.

## Introduction

Sepsis is a leading cause of death in hospitalized patients.^1^ To minimize this risk, early administration of appropriate antimicrobial therapy is required to reduce mortality.^2^^,^^3^ While various sepsis campaigns have focused on early recognition and management, there is a concern that the imperative to administer rapid antimicrobials in septic patients may result in inappropriate antimicrobial use. The early administration of empirical antimicrobial therapy must be balanced with the unintended consequences of antimicrobial use, such as adverse drug effects, antimicrobial- or healthcare-associated infections (e.g. *Clostridioides difficile* infection) and the emergence of drug-resistant microorganisms. Antimicrobial resistance is a serious threat to global public health, with a rapidly increasing health and economic burden.^4^ To balance this risk, guidelines stipulate that empirical antimicrobial therapy in sepsis should be reviewed and de-escalated when possible.^2^^,^^5^ There is increasing evidence that antimicrobial de-escalation for sepsis is a safe strategy with no detrimental impact on mortality.^6–8^ Despite this, approximately 50% of patients with suspected sepsis receive prolonged therapy with unnecessarily broad-spectrum antimicrobials.^9^ At present, antimicrobial stewardship (AMS) interventions have demonstrated improvement in appropriateness of antimicrobial therapy in critically ill patients and those with positive blood cultures, however the benefit of early AMS review in hospital patients with suspected sepsis is less clear.^10–12^ We aimed to determine the impact of early antimicrobial stewardship (AMS) team intervention on optimization of antimicrobial therapy in non-ICU patients treated for suspected sepsis.

## Patients and methods

### Study design and setting

This randomized controlled trial was conducted at the Alfred Hospital (Melbourne, Australia) between February and August 2018. The Alfred Hospital, a university tertiary-care hospital with over 600 beds, is the state referral centre for trauma and burns, as well as bone marrow, heart and lung transplants. Daily AMS rounds were implemented in 2011 across wards outside of the ICU. AMS rounds, undertaken together by one infectious diseases (ID) physician and one AMS pharmacist, consist of a focused review of the patient’s medical record. This combined AMS team provides direct feedback to the prescribers. Prior to this current study, patients were referred for AMS review based on notification from clinical pharmacists for prescriptions of concern (e.g. restricted antimicrobials, prolonged treatment or allergy mismatch) using a web-based antimicrobial approval system (Guidance MS^®^, Melbourne Health) or in patients with a positive blood culture result.^10^^,^^11^ Paper-based medical records were utilized at the time. Formal ID consultation is available at the discretion of the treating medical team. Dedicated formal ID consultation services have been in place for patients admitted to the ICU, Haematology and Transplant Units for over 10 years and these patients are not reviewed by the AMS team.

A Medical Emergency Team (MET) call response is activated within the hospital when a deteriorating patient meets specific clinical criteria (Table S1, available as Supplementary data at *JAC-AMR* Online). Sepsis is considered in all patients who meet MET call criteria, with a specific sepsis prompt included on paper MET call documentation.^13^ The patient is deemed to have suspected sepsis if they meet two or more sepsis criteria and have known or suspected infection (Table S2). Hospital protocol mandates that key sepsis interventions are completed within 1 h of MET calls for suspected sepsis, including administration of broad-spectrum antimicrobial therapy, blood culture collection, lactate measurement and fluid resuscitation.^2^^,^^5^^,^^13^ Empirical antimicrobial therapy is commenced based on the patient’s prior microbiology results in combination with local and national sepsis treatment guidelines. Subsequent management and de-escalation of antimicrobial therapy is at the discretion of the treating team. All MET calls are recorded in the institution’s risk management system (Riskman^®^, Southbank, VIC, Australia) within 24 h.

As part of this study, the AMS service was expanded to identify and include patients with MET calls for suspected sepsis—an important patient cohort who previously did not receive routine early AMS review. All ID physicians and AMS pharmacists who undertook AMS rounds were informed about this service expansion and provided the study protocol.

### Intervention and treatment allocation

Adult patients who had a MET call for suspected sepsis were identified through daily review of MET calls entered into Riskman^®^. Eligible patients who had a MET call for suspected sepsis were randomly assigned to one of two groups. Those patients randomized to the early AMS intervention were reviewed at 48 h following their MET call (or as close as possible to this time). The time period of 48 h was chosen to enable a clearer clinical picture of the patient, and to enable microbiology results to develop. The AMS team reviewed the patient’s medical record, medication chart and pathology results (including relevant microbiology results). Treating teams were contacted to discuss any recommendations to optimize antimicrobial therapy. Eligible study patients who required an AMS review on the weekend were reviewed on the next weekday AMS ward round (eligible within 96 h of the MET call). Those patients randomized to control received standard care, which involved independent management of empirical antimicrobial therapy by the treating team.

Permutated block randomization was performed by computer-generated randomization in 1:1 ratio. Sequentially numbered, opaque envelopes sealed by an independent research assistant concealed the allocation.

### Inclusion and exclusion criteria

Adult patients with their first MET call for suspected sepsis were included, as identified by Riskman^®^ reports. Patients were excluded from this trial if they had a previous MET call for sepsis in the same hospital encounter, pre-existing formal ID consultation involvement, ICU admission within 48 h of the MET call, or a limitation of care order prohibiting active treatment of sepsis. Patients who had already been notified to the AMS team via electronic notification as standard care were also excluded.

### Outcome measures

The primary outcome was proportion of patients receiving appropriate antimicrobial therapy at 24 h after the intervention, or 72 h following the MET call. Antimicrobial appropriateness was assessed using modified Australian National Antimicrobial Prescribing Survey (NAPS) definitions (Table S3).^14^ Antimicrobial therapy was considered ‘appropriate’ if (i) the antimicrobial prescription optimally or adequately followed local or national antimicrobial prescribing guidelines or (ii) the prescribed antimicrobial covered the likely causative or cultured pathogens *and* there was not a narrower spectrum or more appropriate antimicrobial choice available. The ‘appropriate’ definition was modified by removing criteria to indicate appropriateness if it had been reviewed and endorsed by an ID clinician or clinical microbiologist. This modification was required to allow an unbiased assessment of appropriateness for all antimicrobial prescriptions, regardless of involvement by an ID clinician.

Secondary outcome measures included time from MET call to appropriate therapy (in hours), total duration of antimicrobial therapy during the hospital encounter, ICU admission rates following the intervention period, all-cause in-hospital mortality and sepsis-related in-hospital mortality.

A subgroup analysis was performed for patients who met the updated Sepsis-3 definitions (a change in SOFA score of greater than two points).^1^

### Blinding/masking

Appropriateness of antimicrobial therapy was determined by consensus agreement between four independent ID physicians, blinded to allocation. The physicians were provided with a de-identified data collection tool containing relevant clinical information, antimicrobial therapy and microbiology results. Information provided was the same information available to the treating team 72 h following the MET call. Each of the ID physicians adjudicated the assessment of appropriateness individually. In the case of any discrepancy between the assessments, these were discussed in a conference until consensus agreement was achieved.

### Statistical approach

Sample size calculations were based on published Australian data which suggest that approximately 50% of patients with suspected sepsis receive appropriate antimicrobial therapy.^9^

To detect a difference in antimicrobial appropriateness after 72 h between 50% in the control group and 80% in the intervention group with 80% power and alpha of 0.05, 45 patients were required in each arm of the study. A χ^2^ test was used to compare proportions between the intervention and control groups, using an intention to treat approach. For continuous outcomes, a Mann–Whitney *U* test was used to compare medians; time to antimicrobial cessation and time to appropriate antimicrobials were plotted graphically using a Kaplan–Meier (cumulative distribution) graph. Statistical tests were performed using Stata IC 15.1 for Windows (College Station, TX, USA).

### Ethics

The study was approved by the Alfred Human Research Ethics Committee (HREC 587/17) and Monash University Human Research Ethics Committee (MUHREC 12053). It was registered with the Australian New Zealand Clinical Trials Registry (registration number ACTRN12618000116224).

As the AMS team intervention was considered routine care with minimal risk to patients, the requirement for written informed consent was waived by the Ethics Committee. Enrolled patients were provided with a patient information sheet which explained the intervention, use of their clinical information, and how to opt out of the study to withdraw use of their information.

## Results

**Figure 1.**
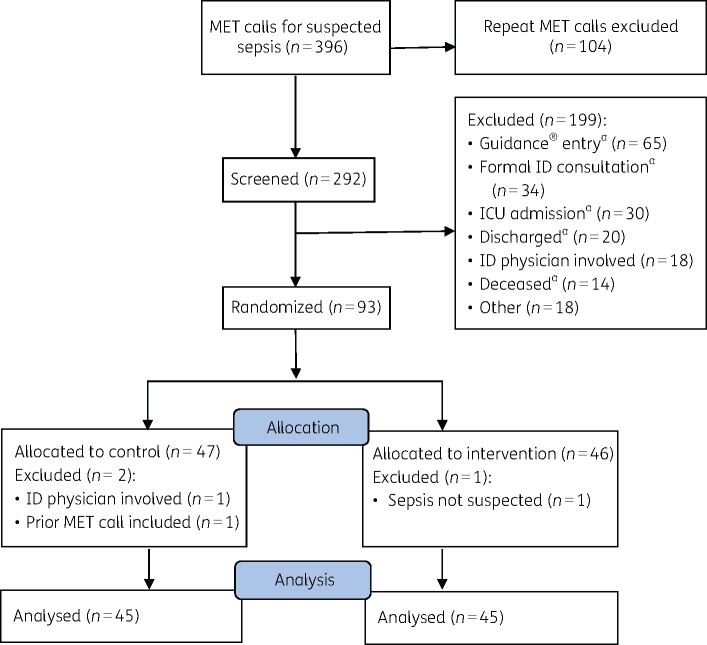
Flow diagram showing patient enrolment. ^a^Within 48 h of MET call.

During the 7 month intervention period (February to August 2018), there were 396 MET calls recorded for suspected sepsis. Of these, 292 reflected a patient’s first MET call for suspected sepsis for inclusion in the study. A total of 199 patients were subsequently excluded for reasons shown in Figure 1. Ninety-three patients were initially randomized; three patients were later excluded for missed exclusion criteria prior to analysis (concurrent ID physician involvement; previous MET call included in the study; and MET call not for suspected sepsis). The study enrolled 90 eligible patients; 45 were randomly allocated to the intervention group and 45 to the control group. A flow chart is shown in Figure 1.

### Baseline characteristics

There were no baseline differences between the two groups (Table 1). On subgroup analysis, 22 patients (49%) in the intervention group and 25 patients (56%) in the control group met Sepsis-3 criteria (change in SOFA score greater than 2 points) (*P* = 0.53).

**Table 1.** Baseline characteristics of patients with MET calls for suspected sepsis

Baseline characteristics	Control, *n* = 45	Intervention, *n* = 45	*P* value
Age, median (IQR)	63 (47–75)	67 (57–75)	0.18
Male, *n* (%)	23 (51%)	28 (62%)	0.23
Blood cultures taken at MET call, *n* (%)	39 (87%)	43 (96%)	0.05
Positive blood cultures at MET call, *n* (%)	7 (16%)	6 (13%)	0.76
CoNS	2	1	
* Escherichia coli*	2	1	
* Klebsiella pneumoniae*	1	1	
* Citrobacter freundii*	1	0	
* Enterococcus faecalis*	1	0	
* Staphylococcus epidermidis*	0	2	
* Pseudomonas aeruginosa*	0	1	
Presumed source of sepsis, *n* (%)			
respiratory	19 (42%)	21 (47%)	0.83
urine	6 (13%)	7 (16%)	1.00
febrile neutropenia	5 (11%)	5 (11%)	1.00
other	15 (33%)	12 (27%)	0.65
Antimicrobials given at MET call, *n* (%)	43 (96%)	44 (98%)	0.58
Sepsis criteria^a^, *n* (%)	25 (56%)	22 (49%)	0.53
Septic shock criteria^b^, *n* (%)	5 (11%)	3 (7%)	0.46

a Defined as a change in SOFA score >2 points.

b Defined as refractory hypotension and lactate >2 mmol/L.

### Primary outcome

Seventy-two hours following a MET call for suspected sepsis a higher proportion of patents in the intervention group were assessed as having appropriate antimicrobial therapy compared with the control group (67% versus 44%, *P* = 0.03) (Table 2).

**Table 2.** Appropriateness of antimicrobial therapy at 72 h after MET calls for suspected sepsis

Appropriateness at 72 h after MET call	Control, *n* = 45	Intervention, *n* = 45	*P* value
Patients on appropriate therapy, *n* (%)	20 (44%)	30 (67%)	0.03
optimal^a^	13 (29 %)	21 (47%)	0.08
adequate^a^	7 (16%)	9 (20%)	0.58
Patients on inappropriate therapy, *n* (%)	25 (55%)	15 (33%)	0.03
suboptimal^a^	19 (42%)	12 (27%)	0.12
inadequate^a^	6 (13%)	3 (7%)	0.29

a Defined as per modified NAPS criteria for appropriateness.^14^

In a subgroup analysis of patients who met Sepsis-3 criteria, the difference in the primary outcome was even greater [15 of 22 (68%) in the intervention group versus 9 of 25 (36%) in the control group, *P* = 0.03]. In patients who did not meet Sepsis-3 criteria, the difference in the primary outcome was not statistically significant [15 of 25 (65%) in the intervention group versus 11 of 20 (55%) in the control group, *P* = 0.49]. In patients without bacteraemia 15 of 38 (39%) of patients in the control group compared with 26 of 39 (66%) in the intervention group were administered appropriate antimicrobial therapy at 72 h (*P* = 0.03). In patients with bacteraemia, there was no difference in the proportion of patients on appropriate therapy [5 of 7 (71%) in the control group versus 4 of 6 (66%) in the intervention group, *P* = 0.85].

### Secondary outcomes

**Figure 2.**
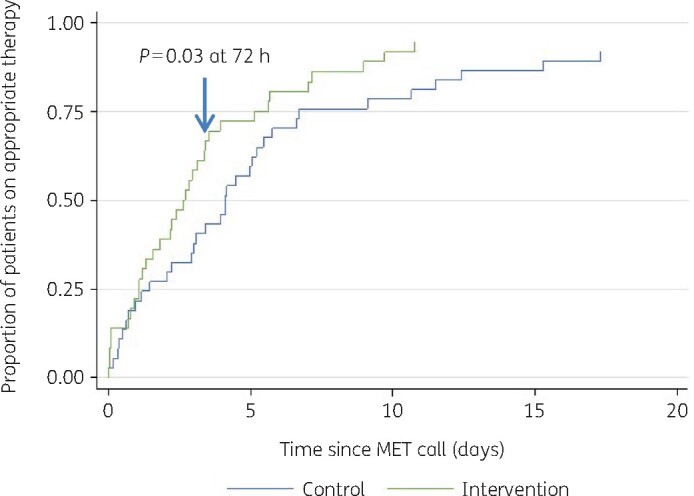
Cumulative distribution graph showing time to appropriate therapy.

The median time from MET call to appropriate antimicrobial therapy was shorter in the intervention group than the control group (43 versus 74 h, *P* = 0.19) (Figure 2). The median duration of total antimicrobial therapy was 8.7 days (209 h) in the intervention group compared with 10.7 days (257 h) in the control group (*P* = 0.39) (Table 3). Overall, 16 patients (18%) were admitted to ICU after the intervention period. There was no difference in ICU admission rates between the intervention (6 of 45; 13%) and control groups (10 of 45; 22%) (*P* = 0.27), or in in-hospital mortality rates (5 of 45; 11% in both groups).

**Table 3.** Clinical outcomes of patients with MET calls for suspected sepsis

Clinical outcomes	Control, *n* = 45	Intervention, *n* = 45	*P* value
Duration of antimicrobial therapy, days, median (IQR)	10.7 (6.0–13.6)	8.7 (5.4–12.9)	0.39
ICU admission post-intervention period, *n* (%)	10 (22%)	6 (13%)	0.27
ICU admission post-intervention period due to sepsis, *n* (%)	8 (18%)	6 (13%)	0.56
ICU LOS, days, median (IQR)	1.6 (1.3–2.0)	1.1 (0.8–3.0)	0.52
All-cause in-hospital mortality, *n* (%)	5 (11%)	5 (11%)	1.00
Sepsis-related in-hospital mortality, *n* (%)	4 (9%)	3 (7%)	0.71

### Antimicrobial prescribing

The most common empirical antimicrobial regimens prescribed at MET calls for suspected sepsis in all patients were piperacillin/tazobactam plus vancomycin (with or without another agent) (21%), piperacillin/tazobactam monotherapy (20%) and meropenem (with or without another agent) (16%) (Table 4). Other agents commonly co-administered included a fluoroquinolone, macrolide, aminoglycoside or sulfamethoxazole/trimethoprim. Approximately half of the enrolled patients (51% in the intervention and 53% in the control group) were prescribed piperacillin/tazobactam at the MET call (in any regimen, not limited to those above).

**Table 4.** Most commonly prescribed antimicrobial regimens

	Control, *n* = 45	Intervention, *n* = 45	*P* value
At time of MET call			
piperacillin/tazobactam plus vancomycin ± other	12 (27%)	7 (15%)	0.20
piperacillin/tazobactam monotherapy	6 (13%)	12 (27%)	0.11
meropenem ± other	7 (16%)	7 (15%)	1.00
piperacillin/tazobactam plus other	7 (16%)	4 (9%)	0.33
other^a^	13 (29%)	15 (33%)	0.65
72 h post MET call			
piperacillin/tazobactam monotherapy	10 (22%)	9 (20%)	0.80
meropenem ± other	8 (18%)	8 (18%)	1.00
piperacillin/tazobactam plus vancomycin± other	4 (9%)	5 (11%)	0.73
piperacillin/tazobactam plus other	4 (9%)	0	0.04
other^a^	15 (33%)	18 (40%)	0.51
no antimicrobial therapy	4 (9%)	5 (11%)	0.73

a Various unique antimicrobial regimens.

### AMS recommendations and acceptance

In the intervention group, the AMS team made 27 recommendations in 25 patients; 74% of these recommendations were accepted by the treating team within 48 h (Table 5). The reasons for not accepting AMS recommendations were not documented. In the remaining 20 patients in the intervention group, the AMS team endorsed the prescribed antimicrobial therapy approach; in 5 patients (of 45; 11%) in whom sepsis had subsequently been excluded, empirical antimicrobial therapy had been ceased prior to the AMS team review and in 15 patients (of 45; 33%) antimicrobial therapy was considered to be appropriate by the AMS team. This is compared with four patients (of 45; 9%) in the control group who had antimicrobial therapy ceased by 72 h.

**Table 5.** Acceptance of AMS recommendations in the intervention group

AMS recommendations	Number of recommendations	Number of recommendations accepted (%)
Total	27	20 (74%)
Discontinue antimicrobial	8	5 (63%)
Switch to oral therapy	6	5 (83%)
Decrease spectrum (de-escalate)	5	2 (40%)
Change antimicrobial to an alternative	3	3 (100%)
Change antimicrobial dose	2	2 (100%)
Recommend formal ID consultation	2	2 (100%)
Initiate new antimicrobial	1	1 (100%)

Piperacillin/tazobactam use was reduced from 51% to 33% in the intervention group and from 53% to 40% in the control group after 72 h (*P* = 0.66). Vancomycin prescribing was reduced from 33% at the time of MET call in both groups, to 16% and 31% (*P* = 0.08) in the control and intervention groups respectively after 72 h. Meropenem use was identical in both groups at the time of MET call (16%) and after 72 h (18%). At 72 h after the MET call, 47% (21/45) of antimicrobial prescriptions in the intervention group were targeted therapy against available microbiological results. In the control group, 36% (16/45) of antimicrobial prescriptions were targeted.

The majority of the AMS reviews (63%) were conducted within 48 h of the MET call, and the median time to review was 2.4 days (57.6 h). AMS service interruptions on the weekend were the most common reason for delayed AMS team reviews. In those patients who received AMS review within the recommended time period, 72% received appropriate antimicrobial therapy at 72 h following the MET call.

## Discussion

In patients who had a MET call for suspected sepsis, early targeted review by an antimicrobial stewardship team resulted in a greater proportion of patients receiving appropriate antimicrobial therapy at 72 h. To our knowledge this is the first randomized control trial assessing the impact of an AMS team intervention on antimicrobial appropriateness for patients with suspected sepsis in a non-ICU setting.

The findings of our study were similar to that observed in the interventional, non-randomized controlled study by Burston *et al*.^9^ The study by Burston *et al*., undertaken in another Australian tertiary care referral hospital, demonstrated a 24% improvement in appropriateness of antimicrobial therapy for patients admitted to non-ICU wards who were identified via a local sepsis pathway.

In a subgroup analysis, Burston and colleagues^9^ found that a difference in antimicrobial appropriateness only occurred in patients who did not have a diagnosis of sepsis (using international Sepsis-3 criteria). The authors hypothesized that this may reflect over-diagnosis of sepsis in hospital sepsis pathways such as theirs, identifying a specific role for AMS intervention in this setting. Conversely, our study demonstrated the greatest impact of AMS intervention in patients *with* a sepsis diagnosis, which may reflect the complexity of patients enrolled in our study. Other studies have documented that early antimicrobial de-escalation is possible in up to 70% of patients following a diagnosis of suspected sepsis.^6^^,^^7^^,^^15–18^ In our study, antimicrobial therapy was safely de-escalated in 30% of patients receiving AMS team intervention, without any detrimental impact on patient outcomes.

In addition to the improvement in appropriateness of antimicrobial therapy, the median time to appropriate therapy was also reduced in the intervention group. As the AMS review was conducted at a standard time each day, the time from individual MET call to AMS review was not always 48 h in each case. Instead, the AMS review was conducted as close as possible and often sooner, to ensure timely intervention and patient care. This likely explains why the median time to appropriate therapy in the intervention group (43 h) is shorter than the suggested 48 h intervention timepoint. Another contributing factor is that 44% of patients in this group (20/45) were already receiving appropriate therapy by the time of intervention, as endorsed by the AMS team.

The reason for non-acceptance of AMS team recommendations was not documented in this study. However, other observational studies have highlighted reluctance to de-escalate therapy in severely ill patients or when a patient is clinically improving as barriers to de-escalation.^6^^,^^16^^,^^17^ The inclusion of an ID physician in the AMS team to assist decision-making in these complex patients may help facilitate de-escalation, as observed in our study. Salahuddin *et al.*^18^ investigated the determinants of de-escalation failure in ICU patients in Saudi Arabia with a diagnosis of sepsis or septic shock. They identified de-escalation rates of 48%, highlighting a number of predictors for their lower de-escalation rates. These predictors include greater organ dysfunction scores, concurrent haematological malignancy and isolation of fungal species or drug-resistant bacteria. Whilst our study excluded ICU patients, these predictors represent an area of focus for AMS teams working in this space.

The all-cause in-hospital mortality rate for patients who had a MET call for suspected sepsis was 11% in our study, which is lower than other reports of inpatient sepsis mortality (15%–35%).^2^^,^^3^ The mortality rate in this study was likely to be lower than other studies due to the inclusion and exclusion criteria; patients who were receiving palliative care, admitted to ICU or died within 48 h of the MET call were excluded from this study.

There were a number of limitations with our study. This study excluded patients who were admitted to the ICU within 48 h of the MET call, as they already had daily input from an ID physician while in the ICU. By excluding these patients, the illness severity of patients in our study may have been lower than has been reported in other studies. Identification of patients with suspected sepsis through our MET call recording system may have missed patients where deterioration was not initially attributed to infection, and this will have omitted them from inclusion in our study. This study relied on existing MET call processes to identify deteriorating patients, however the results of this study could be generalized to other hospitals where other processes are used to identify clinical deterioration. Further limitations include the exclusion of patients admitted under the Infectious Diseases Unit and the availability of the AMS team on weekdays only.

In conclusion, our study demonstrates that in patients who have a MET call for suspected sepsis, AMS team review improves appropriateness of antimicrobial therapy by 23%. By prioritizing this important cohort, AMS teams are able to provide a balanced approach to support early antimicrobial de-escalation and optimization for patients with suspected sepsis.

## Supplementary Material

dlab097_Supplementary_Data
