# Supplementary material for: Early antimicrobial stewardship team intervention on appropriateness of antimicrobial therapy in suspected sepsis: a randomized controlled trial
Source: JAC Antimicrob Resist. 2021 Aug 27;3(3):dlab097. doi: 10.1093/jacamr/dlab097 (PMC8390781; doi:10.1093/jacamr/dlab097)

**Supplementary data**

**Table S1** Alfred Health MET call criteria

| **Vital sign** | **MET call criteria** |
| --- | --- |
| Airway | Threatened |
| Respiratory rate | ≤ 6 breaths/min OR ≥ 36 breaths/min |
| Oxygen saturations | ≤ 90% |
| Systolic blood pressure | ≤ 90 mmHg OR ≥ 200 mmHg |
| Heart rate | ≤ 40 beats/min OR ≥ 140 beats/min |
| Conscious state | - Any unexpected decrease in level of consciousness - Fall in Glasgow Coma Scale (GCS) >2 points - Seizures |
| Other | - Serious concern or uncontrolled pain |

**Table S2.** Alfred Health sepsis criteria

Sepsis should be considered as a cause for deterioration if a patient meets two or more sepsis criteria and has known or suspected infection.

| **Vital sign** | **Sepsis criteria** |
| --- | --- |
| Core temperature (via temporal artery thermometer) | < 36.4˚C or > 38.4˚C OR |
| Oral or equivalent temperature | < 36˚C or > 38˚C |
| Heart rate | > 95 beats/min |
| Respiratory rate | > 22 bpm |
| Systolic blood pressure | < 100 mmHg |
| White cell count | < 4 or >12 x 10^9^/L |
| Change in mental status | - |

**Table S3.** National Antimicrobial Prescribing Survey appropriateness definitions


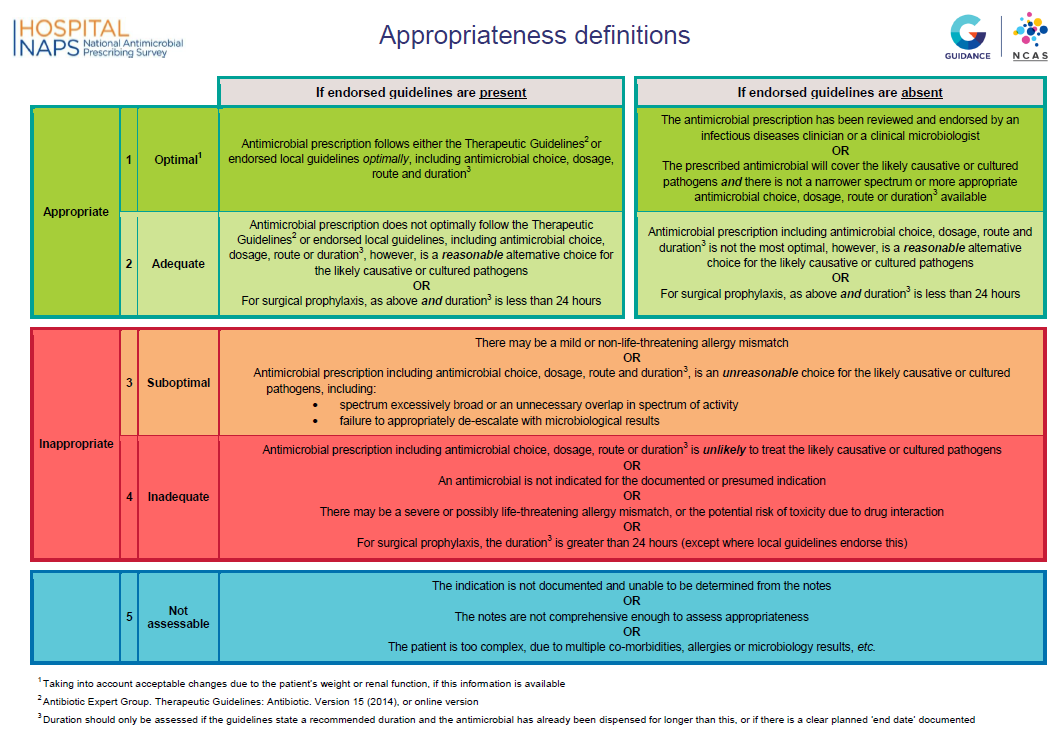

Supplement: dlab097_Supplementary_Data [file dlab097_supplementary_data.docx]
